# Gender-specific link between sleep quality and body composition components: a cross-sectional study on the elderly

**DOI:** 10.1038/s41598-024-58801-5

**Published:** 2024-04-06

**Authors:** Ali Kohanmoo, Asma Kazemi, Morteza Zare, Masoumeh Akhlaghi

**Affiliations:** 1 Department of Community Nutrition, School of Nutrition and Food Sciences, Shiraz University of Medical Sciences, Razi Blvd, Shiraz, 7153675541 Iran; 2 School of Nutrition and Food Sciences, Nutrition Research Center, Shiraz University of Medical Sciences, Shiraz, Iran; 3 Department of Clinical Nutrition, School of Nutrition and Food Sciences, Shiraz University of Medical Sciences, Shiraz, Iran

**Keywords:** Elderly, Sleep quality, Body composition, Skeletal muscle, Body fat percentage, Obesity, Gender, Risk factors, Obesity, Disease prevention, Nutrition, Weight management

## Abstract

Sleep duration has been associated with overweight/obesity. Since sleep quality and body composition alter during aging, we conducted this study to determine if sleep quality is linked to body composition components in elderly people. This is a cross-sectional study conducted on 305 Iranian community-dwelling elderly aged ≥ 65 years. Sleep quality and body composition components were evaluated using Pittsburgh sleep quality index and bioelectric impedance analysis, respectively. The association of sleep quality and body composition components was examined using linear regression analysis. The prevalence of poor sleep quality and overweight/obesity was 48.9% and 54.4% in men and 77.0% and 79.3% in women, respectively. Women had significantly higher scores in most PSQI items than men, indicating their worse sleep quality compared to men. Women also had significantly higher body mass index (BMI), body fat percentage, and visceral adipose tissue and lower skeletal muscle and fat-free mass percentages than men. In the adjusted regression model, men showed positive associations between the third tertile of poor sleep quality and BMI (B = 1.35; 95% CI 0.08–2.61) and waist circumference (B = 4.14; 95% CI 0.39–7.89), but they did not demonstrate an association between sleep quality and body composition components. In the adjusted regression model for women, there were positive associations for BMI (B = 1.21; 95% CI 0.34–2.07), waist circumference (B = 2.95; 95% CI 0.99–4.91), body fat percentage (B = 2.75; 95% CI 1.06–4.45), and visceral adipose tissue (B = 7.80; 95% CI 1.73–13.87); also there were negative associations for skeletal muscle (B =  − 1.40; 95% CI − 2.39 – – 0.41) and fat-free mass (B =  − 2.76; 95% CI − 4.46 – –1.07) percentages. Except for waist circumference, other variables differed between men and women (P < 0.001). Weight management, prevention of muscle wasting, and improvement of sleep quality should be considered in a consortium when designing healthcare strategies for the elderly.

## Introduction

Sleep is a critical aspect of the biological life of humankind^1^. It is necessary for replenishing the energy and alertness for everyday activities and maintaining homeostasis, metabolism, and proper function of the brain and other organs of the body^2–4^. Not only sleep quantity, but also its quality has profound effects on our health^5^. Investigations in different parts of the world have shown that sleep deprivation is prevalent even among healthy individuals^6^. Reports show that adults have an average of 6.8 h sleep in weeknights and 7.8 h on weekends; 62% of adults do not feel they are getting enough sleep^7^. Poor or inadequate sleep has been associated with higher risks of cardiovascular diseases, depression, irritability, Alzheimer’s disease, fall and bone fractures, and chronic pain^3^.

The rate of aging is on the rise worldwide, and Iran is no exception. In this country, the speed of aging is one of the fastest in the world and more than 22% of Iranians are predicted to be over 65 years in 2050^8^. Sleep disorders are common among the elderly^9^. Aging is associated with difficulties in falling asleep, staying asleep, and having a deep sleep^9^. Studies in Iran have demonstrated overall poor sleep quality in older adults^10^. Therefore, it seems beneficial to assess the sleep status alongside other health-related factors in this population.

Obesity, especially abdominal obesity, is related to major non-communicable diseases such as hypertension, type 2 diabetes, and cardiovascular disease and a decline in disease-free years in the elderly^11,12^. Not only obesity, but also undesirably altered body composition measures such as reduced muscle mass and elevated body fat are of concern; alterations that gradually occur with aging^13^. Compared to men, women possess higher rates of obesity and fat mass^14,15^. They also encounter a higher rate of poor sleep quality^16,17^.

Several investigations have indicated an inverse association between sleep duration/quality and common obesity markers, such as body mass index (BMI) and waist circumference^18–22^. However, body composition greatly changes during aging^23^, and is a better estimator of disease risk than obesity markers^24^. In recent years, the relationship between sleep duration/quality and body composition components has been noted by researchers^25,26^ but the influence of gender on such a relationship has not been extensively investigated. Given that body composition is remarkably different between men and women, we questioned whether the relationship between sleep quality and body composition components, such as fat and muscle mass, differs between genders. This relationship is particularly critical for muscle mass, as muscle wasting is one of the major problems of people in old age, increasing the risk of falls and fractures in this population. Women generally possess less muscle mass and bone mineral density than men^27^; so the mentioned relationship may be more crucial for women. Exploring such relationships may help design strategies to attenuate muscle loss and fat accumulation during aging.

## Methods

### Study design

The current cross-sectional study was conducted on 305 elderly people in Shiraz, Iran, from November 2021 to April 2022. The sample size was calculated based on previous investigations^25^ using a correlation coefficient of 0.2 for the association of sleep quality and fat mass percentage, a design effect of 1.5, power of 80%, and significance level of 0.05. Based on the results of linear regression analysis for body fat percentage and sleep quality, the power was estimated 99.5% and 93.2%, for men and women respectively, using R-squared values and the number of covariates in the multiple linear regression model.

The study was conducted according to the guidelines laid down in the Declaration of Helsinki and all procedures involving human subjects. The protocol was approved by the Ethics Committee of Shiraz University of Medical Sciences with the approval code of IR.SUMS.SCHEANUT.REC.1400.002. Written informed consent was obtained from all participants.

### Participants

Elderly people without major diseases were collected from two senior centers under the administration of the General Welfare Organization, and the Abolfazl primary health care center in Shiraz. These samples were selected through simple cluster sampling, in which three clusters were randomly selected out of 11 municipal areas in Shiraz; then one center from each cluster was randomly chosen. Due to the COVID-19 pandemic, participants were recruited by convenience sampling in each center. Thus, all clients who attended the three centers from November 2021 to April 2022 were invited to participate in the study. Inclusion criteria were as follows: age ≥ 65 years, community-dwelling residence in Shiraz, and agreement to participate in the project. People were not included if they were living in nursing or care homes, had surgery, stroke, heart attack, infection, falling, car accident, or hospitalization during the last month, or were afflicted by organ failure, thyroid disorders, serious mental illnesses, cognitive disorders, and dementia. Also, participants using antihistamine and antidepressant medications were excluded.

### Data collection

#### Sleep quality

Sleep quality was evaluated by the Pittsburgh Sleep Quality Index (PSQI) questionnaire, which is a self-rated 18-item questionnaire that assesses sleep quality and sleep disturbances during the past month^28^. The questionnaire has been validated in the elderly Iranian population and has internal consistency and Cronbach's alpha of 0.81 (McDonald’s omega for the original questionnaire = 0.705)^29^. The PSQI score is generated from 7 components: subjective sleep quality, sleep latency, sleep duration, habitual sleep efficiency, sleep disturbances, use of sleeping medication, and daytime dysfunction. Each component of the PSQI questionnaire is scored from 0 to 3, producing a total score of 0 to 21, with higher scores indicating worse sleep quality. A cut-off point > 5 is suggested as poor sleep quality^28^.

#### Anthropometric measures

Body weight was measured with the lightest possible clothes and no shoes by a digital scale (Glamor BS-801, Hitachi, China) to the nearest 0.1 kg. Height was measured by a stadiometer to the nearest 0.1 cm while the participant had shoes off and the head, shoulders, hips and feet were touching the wall. BMI was calculated by dividing weight in kilograms by the square of height in meters. Waist circumference was measured with a non-elastic tape at the mid-point of the distance between the iliac crest and the lowest rib.

#### Body composition

Body composition was analyzed by a portable bioelectric impedance analysis instrument (InBody S10, InBody Co., Seoul, Korea) with four electrodes attaching the wrists and ankles by the standard procedure declared by the manufacturer^30^. Four body composition components were recorded: body fat percentage, skeletal muscle mass percentage, fat-free mass percentage, and visceral adipose tissue area.

#### Physical activity

Physical activity was measured as a confounding factor. As data collection coincided with the COVID-19 pandemic, and the elderly were a high-risk group, we used a single-item questionnaire, which asked: “In the past week/past month, on how many days have you done a total of 30 min or more of physical activity, which was enough to raise your breathing rate. This may include sport, exercise, and brisk walking or cycling for recreation or to get to and from places, but should not include housework or physical activity that may be part of your job”^31^. The questionnaire has been validated in adults and has a modest concurrent validity with the Global Physical Activity Questionnaire (r = 0.53)^31^. Examination of the content validity of the translated questionnaire by an expert panel gave the content validity ratio (CVR) of 0.80 and the content validity index (CVI) of 0.97. Because the questionnaire contained one question, Cronbach’s α could not be determined.

#### Other information

Demographic information, living conditions, medical history, medications, and smoking habits were asked through a researcher-made checklist.

#### Statistical analysis

Statistical analyses were performed by Statistical Package for Social Sciences (SPSS), version 16 (SPSS Inc., Chicago, IL, USA). The normality of data was tested with the Kolmogorov-Simonov test and where needed data were log-transformed before performing statistical tests. The chi-square test was used to compare categorized demographic characteristics between the genders. Since a remarkable difference in sleep quality was observed between the genders, tertiles of sleep quality were determined separately in each gender. Anthropometric and body composition measurements across gender-specific tertiles of sleep quality were compared using one-way ANOVA.

The relationship between anthropometric variables including body composition data and gender-specific tertiles of sleep quality (as the independent variable) was examined using linear regression analysis in the crude model and after adjusting for possible confounders: age, education, marital status, income level, loneliness, physical activity, smoking, self-reported mood disorders, and chronic pain. These factors have been reported to influence sleep duration or quality^32–35^. To calculate the significance of the difference between men and women in the relationships of sleep quality with anthropometric and body composition measurements, linear regression analysis was performed using data from all participants (i.e. men and women), and gender was added among the confounding variables.

Multivariate relationships were examined using one-way multivariate analysis of variance (MANOVA) and Wilks’ Lambda criterion with body fat percentage, skeletal muscle percentage, fat-free mass percentage, and visceral adipose tissue area as dependent variables, gender-specific tertiles of sleep quality as independent variable, and the above-mentioned factors as covariates. For all statistical tests, the level of significance was set at 0.05.

## Results

Overall, 627 individuals aged 65 and older were invited to participate in the study; 290 were not willing to participate, and 32 did not meet the inclusion criteria. Therefore, 305 individuals were included in the analysis. The mean age of the participants was 70.2 ± 5.1 years. They included 92 men (30.2%) and 213 women (69.8%). Of them, 210 (68.9%) were married, 46 (15.1%) had academic education, 254 (83.3%) were living with family, 168 (55.1%) reported to have a low income (related to the first two categories of income listed in Table 1), and 14 (14.1% of men and 0.23% of women) were employed. Demographic characteristics of the participants are presented in Table 1.

**Table 1:** Demographic characteristics of the participants based on gender.

	Men (n = 92)	Women (n = 213)	P value^1^
Age			
65–74.9 y	53 (57.6)	112 (52.6)	0.713
75–84.9 y	23 (25.0)	61 (28.6)	
≥ 85 y	16 (17.4)	40 (18.8)	
Marital status			
Single	10 (10.9)	85 (39.9)	< 0.001
Married	82 (89.1)	128 (60.1)	
Education			
School	71 (77.2)	188 (88.3)	0.013
University	21 (22.8)	25 (11.7)	
Occupation			
Unemployed/housewife	1 (1.1)	159 (74.6)	< 0.001
Employed	13 (14.1)	1 (0.5)	
Retired	78 (84.8)	53 (24.9)	
Living situation			
Alone	4 (4.3)	47 (22.1)	< 0.001
With family	88 (95.7)	166 (77.9)	
Family income			
< 10 million rials	7 (7.6)	17 (8.0)	< 0.001
10–20 million rials	60 (65.2)	84 (39.4)	
20–40 million rials	22 (23.9)	77 (36.2)	
> 40 million rials	3 (3.3)	35 (16.4)	

Data are expressed as number and percentage.

^1^P value was obtained by Chi-square test.

The prevalence of poor sleep quality was significantly higher in women (48.9% in men and 77.0% in women; P < 0.001). Table 2 demonstrates the scores of sleep quality components by gender. PSQI score averaged 6.2 ± 3.6 for men and 8.6 ± 3.7 for women (P < 0.001) (Table 2). Except the time spent in bed, women had significantly higher scores in each component of PSQI: they had shorter sleep duration, longer sleep latency, lower sleep efficiency, more frequent sleep disturbance and sleep medication use, worse daytime dysfunction, lower self-rated sleep quality, and a higher overall PSQI score than men. Fourteen men and 65 women used sleeping pills, 21 (3 men and 18 women) used 1 pill, 19 (2 men and 17 women) used 2 pills, and 39 (9 men and 30 women) used 3 pills during the past month.

**Table 2:** Sleep quality in participants based on gender.

	Men (n = 92)	Women (n = 213)	P value^1^
PSQI score	6.2 ± 3.6	8.6 ± 3.7	< 0.001
Subjective sleep quality score	0.9 ± 0.6	1.2 ± 0.7	0.001
Sleep duration score	1.4 ± 1.0	1.6 ± 1.1	0.050
Sleep latency score	1.2 ± 1.0	1.7 ± 1.0	< 0.001
Habitual sleep efficiency score	0.5 ± 0.9	0.9 ± 1.0	0.001
Sleep disturbance score	1.1 ± 0.5	1.4 ± 0.6	0.001
Use of sleep medication score	0.4 ± 0.9	0.7 ± 1.1	0.009
Daytime dysfunction score	0.8 ± 0.8	1.1 ± 0.9	0.032
Time spent in bed, h	7.0 ± 1.2	7.0 ± 1.1	0.756
Sleep duration, h	6.1 ± 1.2	5.7 ± 1.3	0.025

Values are means ± SD.

^1^Data had abnormal distribution; thus they were log-transformed and compared with independent samples test.

Comparison of anthropometric measures and body composition components between genders revealed that BMI, body fat percentage, and visceral adipose tissue area were significantly higher and skeletal muscle and fat-free mass percentages were significantly lower in women than men (Table 3). The prevalence of excess weight was high among the participants; 54.4% of men and 79.3% of women had overweight/obesity (BMI > 25 kg/m^2^).

**Table 3:** Anthropometric measures and body composition components in the participants based on gender.

	Men (n = 92)			Women (n = 213)			P value^1^
	Minimum	Maximum	Mean ± SD	Minimum	Maximum	Mean ± SD	
BMI, kg/m^2^	17.7	35.6	25.6 ± 3.5	18.9	43.6	28.9 ± 4.7	< 0.001
Waist circumference, cm	72.0	125.0	95.5 ± 10.1	70.0	123.0	97.3 ± 11.0	0.205
Body fat percentage, %	4.0	49.4	22.0 ± 10.6	9.7	55.3	37.1 ± 8.9	< 0.001
Skeletal muscle percentage, %	27.7	54.8	43.5 ± 6.2	23.7	47.3	34.2 ± 5.1	< 0.001
Fat-free mass percentage, %	50.5	96.0	78.0 ± 10.6	44.7	90.2	62.9 ± 8.9	< 0.001
Visceral adipose tissue area, cm^2^	8.8	179.8	67.9 ± 38.9	28.9	187.4	95.9 ± 30.9	< 0.001

^1^Comparisons were performed by independent samples test.

BMI (P ≤ 0.01) and waist circumference (P < 0.01) significantly increased across tertiles (i.e. with worsening) of sleep quality in both men and women (Table 4). However, there was a gender difference in body composition components. For men, no significant pattern was observed across gender-specific sleep quality tertiles, but women demonstrated a significant increase in BF% (P = 0.032) and visceral adipose tissue area (P = 0.010) and a significant decrease in skeletal muscle percentage (P = 0.027) and fat-free mass percentage (P = 0.009) across the tertiles.

**Table 4:** Anthropometric and body composition measures across gender-specific tertiles of PSQI.

	Men				Women			
	Tertile 1 (n = 20)	Tertile 2 (n = 45)	Tertile 3 (n = 27)	P value^1^	Tertile 1 (n = 64)	Tertile 2 (n = 85)	Tertile 3 (n = 64)	P value
BMI, kg/m^2^	23.6 ± 3.21	26.0 ± 3.0	26.4 ± 4.1	0.010	27.5 ± 3.9	28.6 ± 4.8	30.6 ± 4.9	0.001
Waist circumference, cm	89.0 ± 9.1	97.4 ± 8.7	97.6 ± 11.3	0.004	93.9 ± 10.0	96.5 ± 11.2	101.7 ± 10.2	< 0.001
Body fat percentage, %	18.9 ± 9.2	22.2 ± 10.5	24.0 ± 11.4	0.364	34.8 ± 8.1	37.0 ± 9.3	39.6 ± 8.8	0.032
Skeletal muscle percentage, %	45.2 ± 5.9	43.5 ± 6.2	42.2 ± 6.4	0.282	35.4 ± 4.8	34.1 ± 5.2	33.0 ± 5.1	0.021
Fat-free mass percentage, %	81.1 ± 9.2	77.8 ± 10.5	76.0 ± 11.4	0.248	65.2 ± 8.1	63.0 ± 9.3	60.5 ± 8.7	0.007
Visceral adipose tissue area, cm^2^	57.6 ± 27.0	68.8 ± 38.6	72.9 ± 45.3	0.437	87.9 ± 29.9	95.7 ± 31.1	104.4 ± 29.9	0.010

Tertile 1 includes individuals with better sleep quality and tertile 3 is for individuals with poorer sleep quality.

Data are presented as means ± SD.

^1^Comparisons were performed by one-way ANOVA. Data of body fat percentage, skeletal muscle percentage, and fat-free mass percentage in women had skewed distribution and thus they were log-transformed before performing ANOVA.

The association of anthropometric measurements and tertiles of sleep quality was examined with linear regression (Table 5). In men, BMI and waist circumference showed significant positive associations with PSQI in the crude model and after adjusting for confounding factors including age, education, marital status, income level, loneliness, physical activity, smoking, self-reported mood disorders, and chronic pain. In contrast, body composition variables were not associated with gender-specific PSQI tertiles in the crude or adjusted models in men. For women, all associations were significant with the third tertile of PSQI in the crude and adjusted models. Men and women differed in the examined relationships (P < 0.001) except for waist circumference which did not show a significant difference between genders (P = 0.654) (Table 5).

**Table 5:** Linear regression analysis for the association of gender-specific tertiles of PSQI and anthropometric and body composition variables.

	Men				Women				P value for gender difference
	Unadjusted		Adjusted^1^		Unadjusted		Adjusted		
	B coefficient^2^	P value	B coefficient	P value	B coefficient	P value	B coefficient	P value	
BMI									< 0.001
1st tertile PSQI	1		1		1		1		
2nd tertile PSQI	2.46 (0.83, 4.10)	0.004	1.86 (0.72, 3.65)	0.042	1.08 (− 0.37, 2.54)	0.143	0.64 (− 0.92, 2.20)	0.419	
3rd tertile PSQI	1.43 (0.34, 2.52)	0.011	1.35 (0.08, 2.61)	0.037	1.53 (0.76, 2.30)	< 0.001	1.21 (0.34, 2.07)	0.007	
Waist circumference									0.654
1st tertile PSQI	1		1		1		1		
2nd tertile PSQI	8.36 (3.60, 13.11)	0.001	6.50 (1.41, 11.58)	0.013	2.62 (− 0.88, 6.12)	0.141	1.42 (− 2.30, 5.13)	0.452	
3rd tertile PSQI	4.28 (1.17, 7.38)	0.008	4.14 (0.39, 7.89)	0.032	3.89 (2.12, 5.66)	< 0.001	2.95 (0.99, 4.91)	0.004	
Body fat percentage									< 0.001
1st tertile PSQI	1		1		1		1		
2nd tertile PSQI	3.23 (− 2.31, 8.77)	0.248	4.65 (− 0.89, 10.20)	0.098	2.20 (− 0.70, 5.09)	0.136	2.51 (− 0.58, 5.59)	0.111	
3rd tertile PSQI	2.53 (− 0.65, 5.72)	0.116	2.53 (− 1.05, 6.11)	0.161	2.35 (0.87, 3.84)	0.002	2.75 (1.06, 4.45)	0.002	
Skeletal muscle percentage									< 0.001
1st tertile PSQI	1		1		1		1		
2nd tertile PSQI	− 1.70 (− 5.04, 1.65)	0.315	− 2.70 (− 5.99, 0.59)	0.106	− 1.32 (− 2.96, 0.33)	0.115	− 1.49 (− 3.23, 0.25)	0.092	
3rd tertile PSQI	− 1.48 (− 3.35, 0.40)	0.119	− 1.55 (− 3.71, 0.62)	0.156	− 1.19 (− 2.06, − 0.32)	0.008	− 1.40 (− 2.39, − 0.41)	0.006	
Fat-free mass percentage									< 0.001
1st tertile PSQI	1		1		1		1		
2nd tertile PSQI	− 3.22 (− 8.77, 2.33)	0.251	− 4.64 (− 10.20, 0.92)	0.1	− 2.21 (− 5.11, 0.69)	0.134	− 2.53 (− 5.62, 0.57)	0.109	
3rd tertile PSQI	− 2.55 (− 5.74, 0.64)	0.115	− 2.54 (− 6.13, 1.06)	0.161	− 2.36 (− 3.85, − 0.87)	0.002	− 2.76 (− 4.46, − 1.07)	0.002	
Visceral adipose tissue area									< 0.001
1st tertile PSQI	1		1		1		1		
2nd tertile PSQI	11.25 (− 9.20, 31.70)	0.275	15.82 (− 6.27, 37.90)	0.157	7.82 (− 2.34, 17.86)	0.126	6.00 (− 4.86, 16.90)	0.276	
3rd tertile PSQI	7.69 (− 4.61, 19.99)	0.214	6.94 (− 7.81, 21.69)	0.345	8.29 (3.02, 13.57)	0.002	7.80 (1.73, 13.87)	0.012	

Higher PSQI indicates the worst sleep quality.

^1^Adjusted for age, education, marital status, income level, loneliness, physical activity, smoking, self-reported mood disorders, and chronic pain.

^2^Unstandardized B coefficient.

Because there was more than one body composition component, MANOVA test was used to see if there was a significant association between sleep quality tertiles and total body composition data. In agreement with the results of linear regression analysis, men did not show an association between PSQI tertiles and body composition data but women indicated an association in the third tertile of PSQI (Table 6).

**Table 6:** Multivariate analysis of variance examining the association of gender-specific tertiles of PSQI and all body composition data.

	Wilks’ Lambda	Men		Wilks’ Lambda	Women	
		F	P value		F	P value
2nd tertile PSQI						
Unadjusted	0.896	1.65	0.174	0.956	1.63	0.171
Adjusted^1^	0.856	1.10	0.379	0.979	0.589	0.671
3rd tertile PSQI^2^						
Unadjusted	0.911	0.955	0.443	0.880	4.11	0.004
Adjusted	0.798	0.571	0.691	0.873	3.17	0.018

^1^Adjusted for age, education, marital status, income level, loneliness, physical activity, smoking, self-reported mood disorders, and chronic pain.

^2^The third tertile of PSQI indicates the worst sleep quality.

## Discussion

### Main findings

Overall, the results of this study showed considerable differences in the rate of overweight/obesity, body composition, and sleep quality between genders. Women had higher BMI and adiposity and lower skeletal and fat-free mass percentages. They also had shorter sleep duration and poorer overall sleep quality than men. Men showed significant associations between sleep quality and BMI and waist circumference, but not body composition components. Women demonstrated significant associations between all the examined anthropometric and body composition variables and the third PSQI tertile (i.e. the worst sleep quality).

### Prevalence of poor sleep quality

The prevalence of poor sleep quality was high in the participants of this study (68.5%). Previous studies on the Iranian elderly have similarly reported a high rate of poor sleep quality (75%)^36^. However, studies in other parts of the world have reported various rates, ranging from 25% in the USA^37^ to approximately 45% in China^38,39^ and Brazil^40^ to 76% in community-dwelling older adults in Slovenia^41^, but the rates may be higher in nursing home residents (~ 95%)^41,42^. The difference in the sleep quality between countries may be due to differences in the economic status of the nations^43^. Household low income is known as a predictor of poor sleep quality^44^.

### Gender difference in body composition

Women had higher rates of adiposity than men as evidenced by higher BMI, body fat percentage, and visceral adipose tissue area. The rate of obesity based on BMI (i.e. BMI ≥ 30 kg/m^2^) was 10.9% and 38.5% in men and women, respectively. However, based on the ranges of body fat percentage proposed by Gallagher et al. (> 31% and > 43% for obesity in white men and women aged 60–79 years, respectively)^45^, 20.9% and 24.9% of men and women had obesity. It is not clear whether BMI or body fat percentage is a better predictor of obesity, but body fat percentage may be a more reliable indicator because it considers gender in the estimation of obesity. Women possess higher fat masses and are exposed to a higher rate of obesity^14,15,46^. Moreover, women deposit fat mostly in the subcutaneous areas and lower limbs while men store fat mostly around the abdomen, an area associated with the risk of metabolic diseases^47^. This sexual dimorphism is probably mediated through estrogen and androgen receptor-induced gene expression^48^. Women have more fatty acid uptake by adipose tissue, but they also have a higher rate of fat oxidation during exercise^47^. The latter indicates the presence of easily metabolizable fat depots in women which may benefit them in the reproductive process.

Contrary to body fat percentage, women had a lower percentage of skeletal muscle compared to men. With age, muscle mass decreases and fat accumulation increases (possibly because of hormonal alteration and gradual decrease in physical activity), leading to an increased rate of obesity^49^ and sarcopenic obesity^50^ in older adults. The cross-sectional design of this study did not permit seeing the gender difference in fat and muscle mass alteration during the aging process but previous studies have reported that the skeletal muscle declines at a faster rate in men during aging process^51,52^. In this regard, Janssen et al. reported that skeletal muscle percentage in women decreases from 34.1% at the age of 18–29 years to 30.2% at the age of 70 and above (with 11.4% decline), while in men these values are 42.3% and 36.0% (14.9% decline), respectively^52^.

### Gender difference in sleep quality

Women showed a higher prevalence of poor sleep quality (77.0% compared to 48.9% in men). The higher prevalence of poor sleep quality in women has also been reported in previous studies^16,17,53,54^. This gender difference in sleep quality is partly attributed to the higher rate of affective problems such as depression and anxiety in women^55^. Also, lower educational levels, living alone, and low income are among the factors that may affect sleep quality especially in the elderly^56^. However, adjusting for self-reported mood disorders and chronic pain as well as socio-economic (age, education, marital status, income, and loneliness) and lifestyle (physical activity and smoking) factors did not affect the significant difference in sleep quality between genders (data not shown). This finding was also observed in young Australian adults^16^. The reason of higher rate of poor sleep quality in women is not clear based on the results of this and previous investigations and future studies are needed to elucidate the mechanisms involved.

### Poor sleep quality, adiposity, and body composition

Previous investigations have pointed to the inverse association between sleep duration/quality and obesity markers^18–22^. Various explanations have been proposed for this relationship. For instance, during short sleep, changes in eating behaviors such as irregular meal times and frequent snacking occur and contribute to weight gain^57^. In addition, poor sleep may interfere with appetite regulation and satiety signals in the hypothalamus, leading to elevated ghrelin and decreased leptin levels.

In line with our findings in women, a relationship between short sleep and muscle mass depletion in Chinese elderly was reported by Fu et al.^58^ although they did not present the data by gender. The decline in muscle mass may result from hormonal dysregulations during poor sleep. In this regard, Auyeung and colleagues reported that in older men, there is an inverted U-shaped relationship between sleep duration and muscle mass and function, associated also with inverted U-shaped testosterone levels^59^. Even in healthy young males, one-night total sleep deprivation reduces the muscle protein synthesis, increases plasma cortisol, and decreases plasma testosterone levels^60^. In fact, sleep affects testosterone and cortisol levels and sleep deprivation may disrupt their balance and increase muscle proteolysis^61^. Apart from hormonal reasons, poor sleep quality may increase inflammatory markers which may negatively affect muscle anabolism and increase muscle protein breakdown.

### Gender difference in the association of adiposity with sleep quality

There was also a gender difference in the association of obesity and body composition components and sleep quality. Men demonstrated a significant positive association between the markers of general and abdominal obesity and any level of poor sleep quality, while women displayed an association (positive for fat-related components and negative for muscle and fat-free items) between all anthropometric measures and the third tertile of sleep quality.

In contrast to the associations found in this study in both genders (i.e. sleep quality and obesity in men and sleep quality and obesity as well as body composition components in women), studies have mostly found an association between sleep duration/quality and obesity markers in women, but not men^62–68^. For instance, Mamlaki et al. reported that sleep duration or quality was inversely related to BMI and waist circumference in over 65-year-old women but not their male counterparts^64^. Also, in a study on over 1 million participants aged ≥ 30 years, both low and high sleep durations were associated with higher BMI in women (indicating a U-shaped relationship), but in men a longer sleep duration was associated with lower BMI^67^. In line with our results, Patel et al. reported increased BMI and waist circumference in the elderly men and women who slept < 5 h, with greater associations in men, as observed in the current study^22^. The gender-related discrepancies in the results of some studies may be due to differences in the method of measuring sleep (i.e. sleep duration vs. sleep quality), age of participants, degree of adiposity and fat mass in participants of each gender, and control of confounders.

The gender difference which was observed in the association of body composition components and sleep quality in this study may be the result of the difference in the fat and muscle masses between men and women. The magnitude of difference in BMI and waist circumference between genders was not much (~ 13%) but men and women greatly differed in the percentage of fat (~ 69%) and muscle (~ 21%) mass and visceral adipose tissue area (~ 41%); these findings are in the same line with the reports of previous investigators^69,70^. Only few studies have explored gender differences in the association of body composition components with sleep duration/quality. Fan et al. did not find an association between sleep duration and body fat percentage in adult men or women^68^, but two studies found that body fat percentage inversely^70^ and muscle mass positively^69,70^ were associated with sleep duration/quality in males but not females. Due to the very small number of investigations, it is hard to speculate the cause of discrepancies, but differences in sleep duration/quality and the rate of obesity and body fat percentage between genders in each study may be involved. For instance, in the studies of Buchmann et al.^69^ and Nam et al.^70^ higher rates of adiposity were observed in males than females. Other confounding factors such as genetics, psychological conditions, sociodemographic status, diet, and lifestyle might also have differed between studies and caused such discrepancies.

### Strengths and limitations

This study was not without limitations. One of the limitations was the cross-sectional design which did not allow establishing a causal relationship between sleep quality and body composition components. The subjective assessment of sleep quality was not the most accurate way of evaluating sleep, although consistency has been found between studies that have objectively measured sleep quality and those that used self-report measures^71^. Also, the bioelectric impedance analyzer was not the gold standard for the assessment of body composition components. Therefore, the results need to be replicated with more accurate instruments such as dual-energy X-ray absorptiometry. The sample size was not sufficient to generalize the findings to the Iranian elderly population. Also, diabetes was not among the exclusion criteria. However, this work was one of the first studies that tried to explore gender differences in the association between sleep quality and body composition. Considering gender differences in sleep and adiposity, adjusting for potential confounders such as pain and mood disorders, and assessing both sleep duration and quality were among other strengths of this study.

## Conclusions

In conclusion, this study indicated a significant and remarkable difference in the rate of overweight/obesity, percentage of body fat and muscle, and sleep quality between genders. While significant associations were observed between BMI/waist circumference and sleep quality in both genders, only women showed a significant association between body fat and muscle percentages and visceral adipose tissue area on one side and the third tertile of sleep quality (i.e. the worst sleep quality) on the other. Based on these data, a causal relationship between poor sleep quality and increased body fat and decreased muscles cannot be established. However, as body fat and muscle masses are important indicators of health and frailty in the elderly population^72,73^, improvement of sleep quality may lead to greater advantages in the elderly compared to other age groups.

## Data Availability

The datasets used for the current study are available from the corresponding author on reasonable request.
